# The Role of Biomarkers in Lung Cancer Screening

**DOI:** 10.3390/cancers16111980

**Published:** 2024-05-23

**Authors:** Efimia Boutsikou, Georgia Hardavella, Eleni Fili, Aikaterini Bakiri, Stylianos Gaitanakis, Alexandra Kote, Konstantinos Samitas, Ioannis Gkiozos

**Affiliations:** 1 Department of Respiratory Medicine and Oncology, “Theageneio” Anti-Cancer Hospital of Thessaloniki, AL. Simeonidi Str., 54639 Thessaloniki, Greece; efimi_b@yahoo.gr; 2 4th–9th Department of Respiratory Medicine, “Sotiria” Athens’ Chest Diseases Hospital, 152 Mesogeion Av., 11527 Athens, Greece; 3 Health Sciences Library, “Sotiria” Athens’ Chest Diseases Hospital, 152 Mesogeion Av., 11527 Athens, Greece; sot_libr@otenet.gr; 4 1st University Department of Respiratory Medicine, “Sotiria” Athens’ Chest Diseases Hospital, 152 Mesogeion Av., 11527 Athens, Greece; aik.bakiri@gmail.com; 5 Department of Thoracic Surgery, 401 Hellenic Army Hospital, Panagiotis Kanellopoulos Av., 11525 Athens, Greece; sgaitanakis@yahoo.com; 6 6th Department of Respiratory Medicine, “Sotiria” Athens’ Chest Diseases Hospital, 152 Mesogeion Av., 11527 Athens, Greece; akote_1990@hotmail.com; 7 7th Department of Respiratory Medicine, “Sotiria” Athens’ Chest Diseases Hospital, 152 Mesogeion Av., 11527 Athens, Greece; k.samitas@outlook.com; 8 Oncology Unit, 3rd University Department of Internal Medicine, “Sotiria” Athens’ Chest Diseases Hospital, 152 Mesogeion Av., 11527 Athens, Greece; yiannisgk@hotmail.com

**Keywords:** lung cancer, screening, implementation, biomarkers, risk stratification, volatile organic compounds, blood, serum

## Abstract

**Simple Summary:**

Lung Cancer Screening (LCS) is an evolving field presenting variations in its implementation worldwide. This manuscript is delivered by a multidisciplinary Task Force (TF) in LCS. It aims to identify and present existing evidence regarding biomarkers in LCS and form an up-to-date overview of the current evidence.

**Abstract:**

Background: Lung Cancer Screening (LCS) is an evolving field with variations in its implementation in various countries. There are only scarce data from National LCS programs. Aim: We aim to provide an up-to-date overview of the current evidence regarding the use of biomarkers in LCS. Materials and Methods: A multidisciplinary Task Force experts’ panel collaborated and conducted a systematic literature search, followed by screening, review and synthesis of available evidence. Results: Biomarkers in LCS could be used to improve risk stratification in high-risk participants, improve clarification regarding indeterminate lung nodules and avoid overdiagnosis in suspicious lung findings. Currently, there seem to be promising biomarkers (blood/serum/breath) that have been studied in various trials; however, there is still a lack of solid evidence in clinical validation that would pave the way for their integration into LCS programs. Conclusions: Biomarkers are the next logical step in improving the LCS pathway and its efficiency by playing an adjuvant role in a minimally invasive way. National LCS programs and pilot studies should integrate biomarkers to validate their accuracy in real-life LCS participants.

## 1. Introduction

Screening plays an essential role in the early diagnosis of various diseases in high-risk groups. “Prevention is better than treatment”, as Hippocrates said to emphasize the value of prevention for maintaining good health. Early-stage disease detection could translate into more effective patient treatment and improved clinical outcomes. Lung cancer (LC) remains the leading cause of cancer mortality worldwide, and therefore, lung cancer screening (LCS) has an increasingly important potential in early diagnosis [1]. LCS has been established by two large prospective international studies, the National Lung Screening Trial (NLST) and the NELSON trial [2,3]. Both trials have demonstrated that annual low-dose computed tomography (LDCT) screening can reduce LC mortality in high-risk populations, such as heavy smokers, by almost 20%. LDCT is the gold standard screening method, proven to reduce LC mortality. On this background, some European countries have implemented national LCS programs while running pilot LCS studies.

Patient selection for LCS with the use of biomarkers is an important research area as it can offer the possibility to identify high-risk participants not eligible for screening by conventional criteria (age and smoking status), while, at the same time, preventing further investigation in those who do not have malignant disease [4].

Although biomarkers have been thoroughly studied as LC indicators, they have not been included in LCS study protocols to date [5,6,7].

This review aims to identify current evidence regarding the use of biomarkers in LCS programs and their potential utility in affecting clinical outcomes in this context.

## 2. Materials and Methods

A multidisciplinary group of lung cancer experts (respiratory physicians and thoracic surgeons) and a Health Sciences Librarian set a combination of appropriate MeSH keywords and collaborated to identify the relevant literature. Specifically, we searched databases for manuscripts including autoantibodies, complement fragments, microRNA, blood proteins, circulating tumor DNA, DNA methylation and lung cancer detection, diagnosis and screening. All panel members were representatives of the Hellenic Thoracic Society (HTS) or the Hellenic Society of Thoracic, Cardiac and Vascular Surgeons (HSTCVS). PubMed and Cochrane databases were searched, and headings and search results were limited to 1 January 2011–30 June 2023 including publications in English, French and German. The panel independently screened their allocated abstracts and thereafter full papers, performed data extraction and evidence analysis and synthesis. An additional search was conducted for the period 1 July 2023–31 December 2023 with the same methods as stated above. Disagreements were virtually discussed among all team members and a consensus was reached.

Table 1 summarizes all available LCS biomarkers elaborated in this review.

## 3. Results

### 3.1. The Need for Meaningful LCS Biomarkers

**Figure 1:**
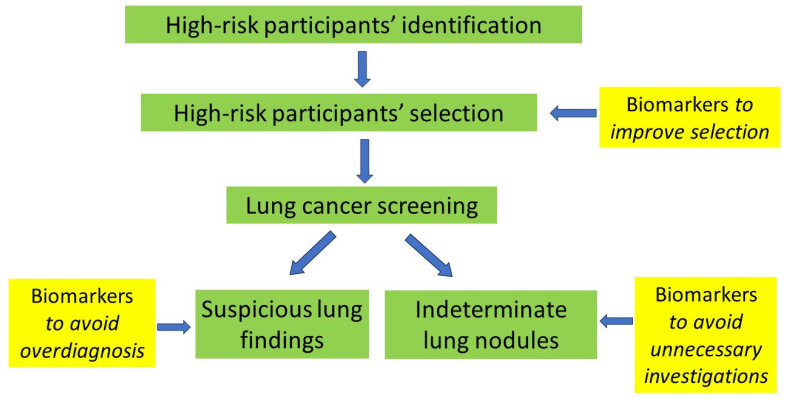
Proposal for biomarkers’ application in LCS pathway. Biomarkers can be used before LCS to improve high-risk participants’ selection as well as during LCS to avoid overdiagnosis and unnecessary investigations.

The optimal LCS biomarker is expected to address three unmet clinical needs: (1) risk stratification to improve the selection of individuals subject to LCS, (2) management of indeterminate nodules for lung cancer detected by LDCT screening and (3) management of suspicious lung findings with the view to reduce overdiagnosis [8] (Figure 1). Sensitivity and cost are two important factors affecting LCS biomarkers’ selection. Limited evidence is available on LCS biomarkers’ sensitivity as well as their potential impact on follow-up studies and biopsies [9,10].

A valuable diagnostic test must be able to classify a patient as either having a disease (sensitivity) or not having a disease (specificity). Additionally, positive and negative predictive values are of equal importance; it is necessary to be able to discern those patients with a positive test who actually have the disease and those with a negative test who are truly disease-free [11]. The use of a risk-predictive biomarker for a disease with low prevalence requires a strong negative predictive value [12]. Furthermore, a clinically useful biomarker should be easily measurable, accurate, reproducible and inexpensive [13].

On this background, the development and clinical validation of an LCS biomarker is necessary to inform LCS programs, and this is challenging. Identifying biomarkers that are both specific to LC and sensitive enough for early detection poses a significant hurdle [14]. Additionally, LC variability in its various subtypes with distinct molecular profiles adds an additional barrier to biomarker research.

The drawbacks of current LCS strategies are the high rate of false positive results [15] and the high prevalence of indeterminate nodules, leading to follow-up diagnostic procedures associated with increased radiation exposure, overdiagnosis and anxiety [16]. There is a vast variety of biomarkers aiming to identify high-risk populations for LCS and complement LDCT’s role with a view to improving its efficiency and diagnostic accuracy. Most data come from prospective cohorts, and the results are promising; therefore, suggesting the use of biomarkers could complement LDCT in LCS. Biomarkers explored until now have been derived mainly from blood, urine or condensate samples. Some of them have synchronous use of imaging, mainly LDCT.

Addressing the challenges in biomarker development requires collaborative efforts across research institutions, healthcare providers and industry partners. Advances in genomics, proteomics and other high-throughput technologies have accelerated the discovery of potential biomarkers. The integration of artificial intelligence and machine learning in analyzing large datasets further enhances our ability to identify patterns and associations that may serve as reliable indicators of lung cancer.

### 3.2. Circulating Blood-Based and Serum-Based Biomarkers

Circulating blood-based and serum-based biomarkers are relatively easy and inexpensive to collect [17].

The EarlyCDT Lung test is a commercially available blood test, based on ELISA principles, that measures a panel of seven tumor-associated autoantibodies: p53, NY-ESO-1, CAGE, GBU4–5, SOX2, HuD, and MAGE A4 [18]. The EarlyCDT Lung test was applied in 235 patients’ samples, and was intended to identify high-risk individuals for LDCT LCS, which is a well-established need. The controls consisted of 266 healthy volunteers. Although promising in terms of its simplicity in sample collection, its performance still remains limited [19]. It presented with high specificity (90.3% [95% CI, 89.5–91.0]), a degree of validation reaching 92% and resulted in a high detection rate of stage I/II LC cases in adults with increased LC risk, as defined by age, smoking history and family history of LC. However, there were several limitations posed by the study design that raised concerns about the validity of the results [4]. Moreover, the effectiveness of the EarlyCDT Lung test in identifying the high-risk subjects in ever-smokers for LCS has not been supported in prospective patient cohorts [20]. The EarlyCDT^®^ Lung test performs best in elderly, late-stage lung cancer patients with a heavy smoking history rather than in identifying high-risk individuals who will benefit from LCS. Therefore, the existing evidence alludes to the insufficient sensitivity of the EarlyCDT^®^ Lung test to be used as part of the inclusion criteria in the LDCT LCS program [21]. Similarly, there is low sensitivity in risk stratification of patients with solid pulmonary nodules [22]. On these grounds, this test does not meet any of the LCS biomarker requirements that would make it a useful tool complementing LCS programs.

A second test, Nodify XL2 (Biodesix, Boulder, CO, USA)—a multiprotein plasma classifier—is also available for the classification of indeterminate pulmonary nodules. This test measures a panel of 11 blood plasma proteins, 5 diagnostic and 6 normalization proteins using a mass spectrometry-based assay. Using a population-based non-small-cell lung cancer prevalence estimate of 23% for 8 to 30 mm indeterminate pulmonary nodules (plasma specimens from 141 subjects with lung nodules were included), the classifier identified likely benign lung nodules with a 90% negative predictive value and a 26% positive predictive value, at 92% sensitivity and 20% specificity, with the lower bound of the classifier’s performance at 70% sensitivity and 48% specificity [23]. The current performance is not favorable for its use as a complementary tool in an LCS program.

### 3.3. RNA-Based Biomarkers

Different circulating RNA species (microRNA (miRNA); piwi-interacting RNAs (piRNAs); transfer RNAs (tRNAs); small nucleolar RNAs (snoRNAs); and small nuclear RNAs (snRNAs)) were identified in human serum [24]. Circulating microRNAs (c-miRNAs) are predominant in the literature, and their remarkable stability in harsh conditions and resistance to circulating RNAses make them ideal candidates for developing lung cancer biomarkers [25].

An miR test is a serum-based miRNA test that measures a signature of 13 miRNAs: miR-92a-3p, miR-30b-5p, miR-191-5p, miR-484, miR-328-3p, miR-30c-5p, miR-374a-5p, let-7d-5p, miR-331-3p, miR-29a-3p, miR-148a-3p, miR-223-3p, and miR-140-5. Montani et al. evaluated the diagnostic performance of an miR test in high-risk individuals (heavy smokers, older than age 50 years, n = 1115) enrolled in the Continuous Observation of Smoking Subjects (COSMOS) LCS program. The overall accuracy, sensitivity and specificity of the miR test were 74.9%, 77.8% and 74.8%, respectively [26].

The miR test is a non-invasive screening method that utilizes the unique expression patterns of miRNAs in biofluids, such as blood or sputum, to detect early-stage lung cancer [27]. By analyzing miRNA profiles associated with lung cancer initiation and progression, the miR test provides a sensitive and specific tool for identifying individuals at high risk, facilitating timely intervention and improved patient outcomes.

### 3.4. miRNA

MicroRNAs (miRNAs) are noncoding and stable RNA fragments regulating gene expression post-transcriptionally. They have emerged as compelling candidates for enhancing LCS programs, offering the potential for early detection, non-invasiveness and improved specificity [28]. Although this does not meet LCS biomarker requirements, it paves the way for further thoughts within the early diagnosis context. In a recent study, a 14-miRNA set distinguished early-stage LC patients with symptoms from individuals without LC, with encouraging accuracy, sensitivity and specificity (95.9%, 76.3% and 97.5%, respectively) [29].

Although this alludes to promising potential in LCS, challenges remain in the way. miRNAs should be integrated into large-scale multicenter LCS studies to establish their reliability and reproducibility, allowing for inclusion in LCS strategies. The standardization of methodologies for miRNA detection and the validation of results across diverse populations are essential steps prior to LCS trials and clinical implementation.

### 3.5. Circulating Tumor Cells (CTCs) for LCS

CTCs are tumor cells in transit in the circulatory system [25]. They originate from primary and secondary tumor sites and are endowed with the molecular features needed to overcome some of the numerous and challenging steps of the metastatic cascade, including intravasation, survival in the blood microenvironment and dissemination to distant organs [30].

The presence of CTCs was shown to anticipate the radiological diagnosis of stage I NSCLC [31], thus leading to an increasing interest around the diagnostic role of CTCs and their implementation as a possible biomarker in LCS programs.

CTC detection for LC diagnosis was found to be promising in initial and explorative studies by Hofman and colleagues [32]. However, the diagnostic accuracy of CTCs detected by ISET^®^ technology in 614 subjects, predominantly men (437 [71%]), aged 65·1 years (SD 6·5), and heavy smokers, is quite low (~26%) and therefore discouraging [33].

Other serum metabolites derived from gas chromatography coupled with mass spectrometry have been used to distinguish individuals with early detected LC from healthy participants of Polish LCS. The majority of the differentiating components were downregulated in cancer samples, including amino acids, carboxylic acids and tocopherols, whereas benzaldehyde was the only compound significantly upregulated. A classifier including nine serum metabolites allowed the separation of cancer and control samples with 100% sensitivity and 95% specificity. The limitations of the study are that it is a cross sectional study, and it includes a very small patient size. This signature of serum metabolites deserves further investigation to be established [34].

LC-related tumor markers either alone or in combination (CEA, CA125, CY211, NSE and SCC) have also been studied. The optimal biomarker combination was CEA  +  CA125, with a sensitivity of 0.667 and a specificity of 0.877, and it was reported to have an improved performance versus multi-marker combinations [35].

Small extracellular vesicles (sEVs) circulating in human biofluids, potential sources of cancer biomarkers, appeared recently as an attractive type of “liquid biopsy” [36]. sEVs are nanosized vesicles (30–150 nm) secreted by various cell types, including cancer cells, into the extracellular environment. These vesicles contain a cargo of proteins, lipids, nucleic acids and other biomolecules reflective of their cell of origin, making them valuable sources of molecular information. In the context of lung cancer, sEVs shed light on tumor biology, progression and response to treatment, offering insights into the disease’s molecular landscape [37].

Based on these findings, M. Smolarz et al. compared the lipid profiles of serum-derived sEVs from three groups of LCS participants: individuals without pulmonary alterations (n = 81), individuals with benign lung nodules (n = 81) and patients with screening-detected lung cancer (n = 81). Although a few lipids whose levels in serum-derived sEVs were different between the three groups of participants in the LCS study, the data obtained do not support the concept of using the metabolites present in serum-derived “total” sEV as biomarkers for early lung cancer detection [38].

### 3.6. Breath Biomarkers

Given the limitations and relatively high failure rate of serum cancer biomarkers, alternative biological samples have been explored as an alternative in LCS [39]. A promising biofluid that is rarely used for diagnostic purposes is exhaled breath condensate (EBC), the composition of which has been inadequately studied. The EBC is a promising matrix due to the easiness of sampling, real-time analysis and non-invasive characteristics.

Breath analysis has become a research focus in the field of respiratory disease diagnosis due to its noninvasiveness and real-time analysis. The principal component in breath is water vapor, and the remaining parts include volatile organic compounds (VOCs) and nonvolatile matters dissolved in water or contained in exhaled aerosol particles.

A miniature e-nose system using 14 gas sensors from 4 different sensor array types could identify relatively specific “breath fingerprints” based on 235 human breath samples (134 healthy controls and 101 cases from lung cancer patients), which could be used to recognize volunteers in different diseased or healthy states. Linear discriminant analysis (LDA) proved to be among the best methods in terms of classification performance, and in combination with the classifier Fuzzy k-NN, it showed the best classification results, which produced greater than 90% sensitivity, specificity and accuracy. The designed e-nose system based on optimized algorithms was low-cost, noninvasive and potentially practicable in screening lung cancers from both healthy people and high-risk populations [40].

Despite this, the large-scale implementation of e-noses still faces challenges due to the limitations of existing gas sensors that need to identify odor categories or quantify odor intensities in non laboratory conditions, i.e., in real-life clinical settings. Artificial intelligence (AI) has assisted in this direction; however, several steps need to be made further [41] to generate new ideas.

### 3.7. Volatile Organic Compounds (VOCs)

The detection of volatile organic compounds (VOCs) in exhaled breath has been a recent approach of great interest [42]. VOCs seem promising, and they exhibit 100% sensitivity, 92.86% specificity and 95.74% accuracy in LC diagnosis [43].

VOCs in exhaled breath samples of LC patients are significantly different from those of patients with benign pulmonary nodules or no LDCT findings at all [44]. This paves the way for a non-invasive method to detect lung cancer and a potential future application in LCS programs; however, clinical validation is yet to be completed. LDCTs used in LCS require appropriate infrastructure and human resources for performing them as well as reporting them. This places LCS in an expensive and sometimes complex pathway. VOCs seem to be a cost-effective alternative, with butyraldehyde, butyric acid and dicyclohexyl ketone ranked in the first three for distinguishing lung cancer patients (n = 156) from healthy participants (n = 193) with 92% accuracy [45]. It seems that VOC measurement in exhaled breath offers an accurate classification for the presence or absence of lung cancer without the need for more invasive methods (e.g., biopsies) or methods requiring radiation exposure (e.g., radiological follow-up of indeterminate lung nodules). Clinical validation is an ongoing theme that requires attention, and it should be addressed in the future.

The clinical application of VOC measurement and assessment in LCS poses several challenges. Study designs, respiration sampling techniques and subsequent data collection and analysis lead to contradictory results [4]. Recent consumption of food and the overall nutritional habits of participants exhibit an effect on VOC samples and their measurement as food consumption affects the results [46]. There is no clear cut-off timeframe to test VOCs after meal consumption to ensure it is not affected by dietary intake. The duration and type of fasting still remain arbitrary in various study protocols [47]. The question arises as to whether future studies should implement fasting prior to VOC collection or whether they should collect data on different dietary consumption to ensure there is a pragmatic future approach to the implementation of VOC measurement in LCS. Another issue raising concerns is the effect of oxidative stress and lipid metabolism on VOC measurement, which can significantly impact the results of VOC measurement from various individuals [48].

## 4. Discussion

LCS is an important intervention to reduce LC-specific and all-cause cancer mortality. However, participant selection is hampered by the narrow selection criteria (age and smoking history), leading to missing a significant number of LC cases. Although the widening of the current selection criteria seems to be the next logical step, this increases false positive rates. LCS risk stratification models seem to have partially covered this gap, where there is still room for improvement with the addition of non-invasive reliable biomarkers that will complement LCS [4]. Biomarkers offer a promising approach to LCS as they may be able to identify people at high risk for LC without concurrently elevated risk for diseases related to age and smoking. Appropriate LCS biomarker selection and application will lead to improvements in risk stratification and false positivity, therefore encouraging high-risk individuals to participate in LCS with an improved LCS acceptance rate [28].

There are still several unmet needs in the LC biomarker field. We are still missing a panel of biomarkers that will complement risk stratification for LCS and appropriate participant selection as well as classify indeterminate pulmonary nodules that pose a major burden on clinical services and resources as well as LCS participants. Despite references in the literature for potential biomarker use in this direction, evidence on clinical validation is lacking, and therefore, these biomarkers have not been applied to date [49]. There is great diversity in the types of patients/participants recruited in these studies, which may affect performance characteristics, and it can be misleading in terms of the true efficiency a biomarker panel may have. Only in some studies have the participants/patients tested constituted a true screening cohort (meeting USPSTF eligibility), but overall, there is a lack of data on racial and ethnic representation, and cancer cases are not weighted toward stages. These matters raise concerns about testing against inappropriately selected cohorts, which can be highly misleading in terms of a method’s potential value to benefit LCS.

Blood/serum-based and breath biomarkers require specific infrastructure for processing and storage, and this comes with an extra cost. Nation-wide implementation of LCS should be accessible to all high-risk participants regardless of geography. The adjuvant use of blood/serum and/or breath biomarkers in combination with the widespread implementation of LCS across all geographical areas poses the challenge of sample collection, storage and processing in centers devoid of such infrastructure. Therefore, biomarker samples will need to be transferred to central hubs for processing, and this would increase LCS costs and also pose the risk of sample damage at transfer. Biomarker testing requires specialized equipment and expertise, making it more suitable for central hubs with relevant infrastructure. Healthcare personnel need to be trained in sample processing and conducting measurements, while an LCS expert team is required to interpret the results in the context of real-life high-risk individuals participating in LCS. The future implementation of LCS biomarkers in clinical practice should be carefully designed to ensure it is performed in centers with expertise and that all LCS participants have equal access to this service regardless of geography. Equal access to an LCS service incorporating biomarker testing can ensure under-represented populations are identified and invited to LCS programs. Improved identification of the target population can potentially reduce disparities in clinical outcomes and improve overall survival rates. Culturally sensitive approaches, along with targeted interventions addressing socioeconomic barriers, are critical for achieving this goal.

The literature identifies the need for the potential use of biomarkers in LCS programs; however, the current evidence does not identify an optimal biomarker to be used in this capacity. The main problem in biomarker studies remains the short period of follow-up and the selection of validation sets, which could be overcome with an appropriate study design [4]. Therefore, there has been no approval of any biomarker for LCS programs, as current evidence does not justify the validity or cost-effectiveness of this intervention. It is of note that only one study clearly states the degree of validation [18], which highlights the unmet need for further clinical validation studies to provide this important information that will inform potential applications in clinical practice. The necessity of biomarkers lies in the fact that a biomarker-based companion diagnostic test would be expected to lower the false positivity rate as well as reduce overdiagnosis and improve the performance of LDCT for LCS. Among all biomarkers studied (Table 1), VOCs present the highest sensitivity and specificity, while tumor markers present the lowest. Even though this is a promising hint, clinical validation is required, as well as further insights into the fragility of samples, their storage and their processing before these are applied widely.

## 5. Conclusions

There is a vast variety of biomarkers aiming to identify high-risk populations for LCS and complement LDCT’s role, with a view to improving its efficiency and diagnostic accuracy. Most data come from prospective cohorts, and the results are promising; therefore, suggesting the use of biomarkers could complement LDCT in LCS. However, further clinical validation is required to lead to future application in daily clinical practice. There is a lack of data suggesting which is the optimal biomarker in LCS. In conclusion, the panel cannot recommend the use of biomarkers in LCS, only in the context of a clinical trial.

### Future Directions

Biomarkers can be promising tools complementing LCS in a minimally invasive way to ensure the following:

1. Optimal participant selection, i.e., identifying high-risk individuals that will, indeed, benefit from LDS. Biomarkers, in this case, will play a complementary role in simple inclusion criteria as well as risk stratification models for LCS.
2. The clarification of the status of indeterminate pulmonary nodules to avoid unnecessary follow-up and investigations that will increase the burden on existing resources and participant anxiety.
3. The clarification of suspicious lung findings with the view to minimizing or even completely avoiding unnecessary investigations and diminishing overdiagnosis.

National LCS programs and pilot studies should integrate biomarkers as part of their clinical research with the view to validating their accuracy in real-life LCS participants.

## Figures and Tables

**Table 1:** LCS biomarkers.

	Sensitivity	Specificity	Positive Predictive Value (PPV)	Negative Predictive Value (NPV)
**EarlyCDT Lung**	54.6 (32.2–75.6)	90.3 (89.6–91.1)	2.0 (1.0–3.5)	99.8 (99.7–99.9)
**Nodify XL2** (Biodesix, Boulder, CO, USA)	92	48	26	90
**MSC algorithm**	87	81	25	99
**Serum metabolites (GC/MS)**	100	95		
**Tumor markers**				
CEA + CA125	75.5	79.1	74.6	79.9
CEA + CY211	76.1	71.8	68.7	78.8
**E-nose** (LDA-Fuzzy 5-NN)	91.58% [90.01%, 93.15%]	91.72% [90.35%, 93.09%]	Not mentioned	Not mentioned
**Volatile organic compounds**	100%	93%	Not mentioned	Not mentioned

## Data Availability

Bibliographical retrieval data are available by the corresponding author and the Hellenic Thoracic Society, the Hellenic Radiological Society and the Hellenic Society of Thoracic, Cardiac and Vascular Surgeons should they be required.
